# Do Calcium Chelators Play a Role in the Removal of Calcium Hydroxide From Root Canals? A Systematic Review of Laboratory Studies

**DOI:** 10.14744/eej.2021.73644

**Published:** 2022-03-22

**Authors:** Nandini SURESH, Aswathi VARGHESE, Sathish SUNDAR, Venkateshbabu NAGENDRABABU, Natanasabapathy VELMURUGAN

**Affiliations:** From the Department of Conservative Dentistry and Endodontics (N.S. 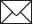 nandini_80@hotmail.com, A.V., S.S., N.V.) Meenakshi Ammal Dental College & Hospital, Meenakshi Academy of Higher Education and Research (MAHER), Tamilnadu, India; Department of Preventive and Restorative Dentistry (V.N.), University of Sharjah, Sharjah, UAE

**Keywords:** Calcium hydroxide, chelation, citric acid, ethylenediaminetetraacetic acid, systematic review

## Abstract

**Objective:**

To identify whether root canal irrigants with calcium chelation ability play a role in the removal of calcium hydroxide (CH) from the root canals when compared to non-chelators.

**Methods:**

The protocol is registered in the Open Science Framework registry (doi 10.17605/OSF.IO/CHG2Q). PubMed, Scopus, Embase, Cochrane Library, ProQuest, Google Scholar, Science direct and open grey databases were searched until March 2021. Laboratory studies comparing the effectiveness of calcium chelators in the removal of CH with non-chelators delivered using needle irrigation, irrigation agitation or instrumentation techniques were included. The quality of included studies was appraised using a modified Joanna Briggs Institute critical appraisal checklist for a randomised clinical trial. Two independent reviewers were involved in study selection, data extraction, appraising the quality of studies. Any disagreements were resolved by a third reviewer.

**Results:**

The current review included 17 studies, with 16 being of "moderate" quality and one of "low" quality. Due to methodological differences within the included studies, quantitative analysis was not performed. Laboratory studies were only included in the current review because no clinical study exists on this topic. Evidence from the review indicates that calcium chelators are superior to non-chelators in the removal of CH when used with needle irrigation, passive ultrasonic irrigation and instrumentation techniques.

**Conclusion:**

Calcium chelators are superior in the removal of CH from the root canal system over non-chelators.

**HIGHLIGHTS**

• Calcium chelators enhance the removal of CH intracanal medicament from the root canal when used with needle irrigation techniques compared to non-calcium chelators.
• Calcium chelators enhance the removal of CH intracanal medicament from the root canal when used with agitation techniques such as passive ultrasonic instrumentation and rotary instrument compared to non-calcium chelators.
• The powder form of CH, which is mixed with sterile saline or distilled water, is more effectively removed with chelators when compared to a non-chelating agent.

## INTRODUCTION

Calcium hydroxide (CH) is a widely used intracanal medicament in the field of endodontics due to its excellent antimicrobial property (1), ability to inhibit osteoclastic activity (2) and to produce a favourable tissue repair response (3). However, remnants of CH in the root canal system hinders the penetration of endodontic sealers into the dentinal tubules (4), which affects the sealing ability of the root canal sealers (5) and increases the apical leakage (6) as well as affects the setting of zinc oxide-eugenol based sealers (7). Goldberg et al. (8) has shown that CH remnants in the root canal affected the sealer penetration into lateral canals. Similarly, a systematic review has concluded that there is a reduction of mechanical property of root dentin when exposed to CH for more than five weeks (9). Hence, the removal of CH before obturation is an important step and proper techniques for removal should be followed.

Methods of removing CH from the root canal system can be divided into 3 broad categories: instrumentation with irrigants (10, 11), irrigants delivered by manual irrigation technique like syringe irrigation, and irrigants delivered by machine-assisted irrigation like passive ultrasonic irrigation (PUI) (12), EndoVac (Discus Dental, CA, USA), (10), RinsEndo (Durr Dental, Bietigheim-Bissingen, Germany) (13). 

The observations from previous studies pertaining to calcium hydroxide removal can be categorised into mainly two parameters a) role of chelators and other root canals irrigants, and b) role of irrigation dynamics. An array of chelators like ethylenediaminetetraacetic acid (EDTA), citric acid and maleic acid have been used to remove CH from the root canal and have been proven to be superior when compared to non-chelating agents in the removal of calcium hydroxide (10, 11). However, irrigants like sodium hypochlorite (NaOCl), distilled water or saline, which does not have a calcium chelating effect, efficiently removes CH when used with various agitation systems (14, 15).

Various irrigation activation systems utilise properties such as acoustic streaming in PUI (12), negative pressure in EndoVac (16), vigorous intracanal fluid agitation in EndoActivator (Advanced Endodontics, CA, USA) (17) and abrasive lattice motion in Self-adjusting file (SAF; ReDent NOVA, Berlin, Germany) (18) to increase the removal of calcium hydroxide from the canals. CH intracanal medicament was found to be effectively removed using PUI from the root canals by different studies (14, 15, 19). Çapar et al. (11) reported that CH removal is improved with the use of calcium chelators with SAF and PUI in comparison to that of a non-chelator. In contrast, Kuga et al. (20) has shown no superiority of calcium chelator over a non-chelator in the removal of CH when used as an adjunct with rotary instruments. However, the exposure to chemicals and irrigation agitation systems can result in increased dentine erosion (21, 22), leading to reduced dentine hardness.

In endodontic literature, the need to use calcium chelators to remove CH remains debatable, resulting in a difficult situation for the clinician in selecting the appropriate irrigant. Hence, the current systematic review was undertaken. Currently, no clinical studies in the literature assess the role of irrigating with calcium chelators in removing calcium hydroxide intracanal medicament. Thus, the evidence is cumulated from laboratory studies with the following objectives: i) to compare the effectiveness of calcium chelators in removing CH to that of non-chelators when delivered with needle irrigation and ii) to compare the effectiveness of calcium chelators in removing CH when delivered with irrigation agitation, instrumentation techniques, and machine-assisted irrigation. 

## MATERIALS AND METHODS

The current systematic review was reported according to the Preferred Reporting Items for Systematic Reviews and Meta-Analyses statement (23) and the protocol of the review was registered in the Open Science Framework registry (Centre for Open Science, osf.io/chg2q/registrations, DOI 10.17605/OSF.IO/CHG2Q).

### Research question

The following research question was developed based on PICOS format: 

PICOS (P -Population, I -Intervention, C -Comparison, O -Outcome, S -Study design).

1. Do calcium chelators (I) have a better ability to remove CH (O) compared to non-calcium chelators (C) when delivered with needle irrigation technique in the extracted human permanent teeth (P) from laboratory-based studies (S)?
2. Do calcium chelators (I) have a better ability to remove CH (O) compared to non-calcium chelators (C) when delivered along with irrigation agitation or instrumentation techniques in the extracted human permanent teeth (P) from laboratory-based studies (S)?

### Literature search

The search was performed in PubMed, Scopus, Embase, the Cochrane Library, ProQuest, Google Scholar, Science direct and open grey from inception until March 2021. The search strategy was developed for each electronic database (Appendix 1). Only articles published in the English language were included in this review. The reference list of the included studies and previously published reviews were additionally searched. Additionally, 3 endodontic journals: the Australian Endodontic Journal, the International Endodontic Journal, and the Journal of Endodontics, were screened up to March 2021 for articles that were not found in the databases. If necessary, the corresponding authors were contacted to obtain missing information. Zotero software (Corporation for Digital Scholarship, Virginia, USA) was used to remove duplicates and organise the identified studies. Two independent reviewers (AV, SS) performed title, abstract and full-text assessment. Any disagreement will be resolved by a third reviewer (NS).

### Eligibility criteria

#### Inclusion criteria

1. Laboratory studies that assessed the efficacy of CH removal using calcium chelator in comparison to a non-chelator in extracted adult human permanent teeth.
2. Studies using any endodontic files and (or) irrigation agitation system and (or) needle irrigation.
3. The removal efficiency of CH was assessed using scanning electron microscope (SEM) image analysis or computed tomography (CT) or cone-beam computed tomography (CBCT).

#### Exclusion criteria

1. Studies not comparing a calcium chelator to a non-chelator in studying the effectiveness of CH removal.
2. Animal studies, studies using artificial resin canals, case reports, case series and reviews.

### Data extraction

The data extraction form was created in an Excel sheet, and the following parameters were extracted from the articles: the surname of the first author, year, country of the first author, interventions, method of assessment and conclusion of the study. Two independent reviewers (AV, NV) performed the data extraction, and any disagreement was resolved by discussion with a third reviewer (NS).

### Quality assessment

The quality assessment of the included studies was performed by two independent reviewers (AV and SS), and disagreements were resolved by consultation with a third reviewer (NS). The Joanna Briggs Institute, Critical Appraisal Checklist for randomized controlled trials (https://jbi.global/critical-appraisal-tools) was modified according to the current systematic review. The checklist was modified to a total of 12 criteria which included items on sample size calculation and standardisation of samples to assess selection bias. The items pertaining to follow up of treatment and trial design were excluded. A score of "1" was given if the criterion was met, and a score of "0" was given if the criterion was not met, unclear or not applicable. The included studies in systematic review were categorised into low (1-2-3-4, 0-33% points); moderate (5-6-7-8, 34-75% points) and high (9-10-11-12, 76-100% points) quality (24). The inter-rater reliability between the two examiners (AV and SS) were calculated by Cohen's kappa coefficient using online software graphpad.com.

## RESULTS

### Study selection

**Figure 1.**
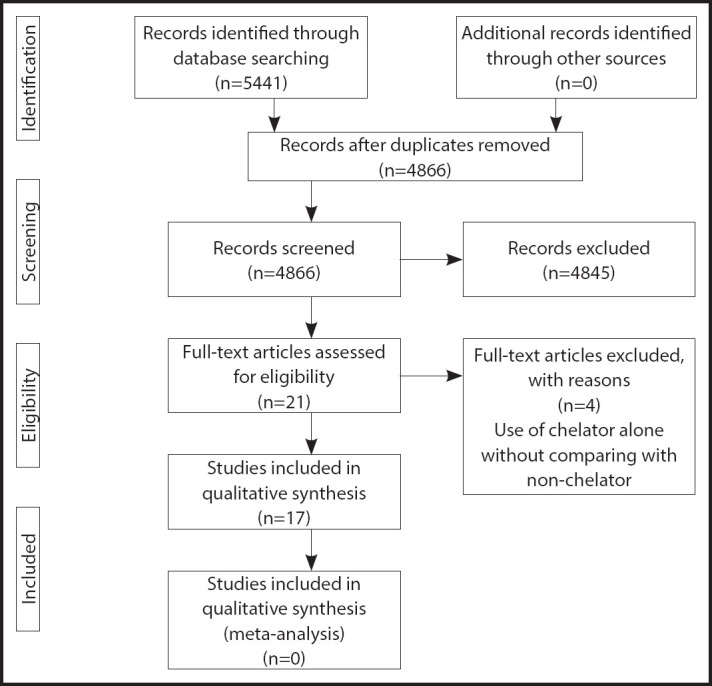
PRISMA flowchart

The literature search process is provided in Figure 1. The initial search retrieved 5441 titles or abstracts from all the electronics databases. Among these, 575 articles were eliminated as duplicates. After reading titles or abstracts, 21 articles were eligible for full text assessment. Among those 4 were studies that do not contain a group that compares with a non-chelating agent and hence were excluded (25-28). Finally, 17 articles were included for the systematic review (10, 11, 20, 29-42). A quantitative analysis (meta-analysis) was not performed because of the substantial heterogeneity of the included articles. 

### Characteristics of the included studies

The characteristics of the included studies are shown in Table 1 and Table 2. Mandibular premolars were the predominantly (10, 11, 32, 33, 36-42) used teeth for the CH removal assessment. The overall number of samples in the included studies ranged between 28 to 160. A total of 5 studies (34, 36, 38, 39, 40) were performed in roots with a curvature that ranged from 5° to 10o and 4 studies (10, 29, 35, 42) have used straight canals, whereas the remaining studies did not mention the curvature used in the studies (11, 20, 30-33, 38). Neelakantan et al. (41) used premolars with oval canals after confirmation with CBCT. EDTA (ranging between 5% to 20%) was employed as a calcium chelator in all the included studies. The other calcium chelators that were used in the studies were citric acid (10%-50%), etidronic acid (18%), chitosan (0.2%), maleic acid (7%), phosphoric acid (37%) and peracetic acid (1%). Based on the qualitative analysis, 12 studies showed various calcium chelators (e.g., EDTA, Smear Clear, citric acid, maleic acid, peracetic acid, Qmix, etidronic acid, chitosan) enhanced the removal of calcium hydroxide intracanal medicament from the root canal when compared to non-calcium chelators (e.g., NaOCl, distilled water) (Tables 1, 2).

**TABLE 1.** Characteristics of the included studies, which shows chelators has a beneficial effect in removal of CH compared to non-chelator

Author, year	Tooth	Assessment method	Group area assessed	Conclusion
Abi-Rached et al. 2014 (10)	Premolar (mand)	Sectioning and SEM (1000x)	Chelators: 17% EDTA (3 mL), 17% EDTA (3 mL)+2% CHX (1 mL) Non chelators: Sterile saline (15 mL+10 mL), Agitation system: MAF+2 Further files Coronal, middle and apical	17% EDTA removed CH+2% CHX better than 17% EDTA+2% CHX combination
Anitha et al. 2012 (35)	Central Incisors (max)	Sectioning and Microscope (12.5x)	Chelators: Smear Clear (17% EDTA) (3 mL), 10% CA (3 mL), 5% EDTA (3 mL) Non chelators: 3% NaOCl (3 mL), Agitation system: PUI Coronal and apical	Smear Clear and 10% CA>5% EDTA and 3% NaOCl (Coronal and Apical levels) More residues in apical groove than in coronal groove
Arslan et al. 2014 (36)	Premolar (mand)	Sectioning and Stereomicroscope (25x) n=48	Chelators:17% EDTA (5 mL), 7% MA (5 mL), 10% CA (5 mL) Non chelators: 1% NaOCl (5 mL), Agitation system: NI Artificial groove in apical region	7% MA=10% CA>17% EDTA=1% NaOCl
Çapar et al. 2014 (11)	Premolar (mand)	Sectioning and Stereomicroscope (30x) n=88	Chelators: 2.5% NaOCl (10 mL)+17% EDTA (10 mL) Non chelators: 2.5% NaOCl (10 mL), Agitation system: NI, SAF, Endovac, PUI Artificial groove in apical region	2.5% NaOCl+17% EDTA>2.5% NaOCl (Only with SAF)
da Silva et al. 2011 (34)	Molars (mand)	Sectioning and SEM (1000x) n=48	Chelators: 17% EDTA (5 mL), 10% CA (5 mL), 37% phosphoric acid (5 mL) Non chelators: 2.5% NaOCl (5 mL), Agitation system: MAF Coronal, middle and apical	37% PA=10% CA=17% EDTA-T>2.5% NaOCl (Coronal and middle third)
Kuştarcı et al. 2016 (38)	Premolar (mand)	Sectioning and Stereomicroscope (6x) n=160	Chelators:17% EDTA (6.5 mL), Qmix (6.5 mL), 1% PAA (6.5 mL) Non chelators: 2.5% NaOCl (6.5 mL), Agitation system: NI, LAI Artificial groove (2-6 mm from apex only)	LAI>NI 17% EDTA=Qmix=1% PAA>2.5% NaOCl (for both LAI and NI)
Li et al. 2010 (32)	Premolar (mand)	Sectioning and SEM (1500x) n=200	Chelators: 2.5% NaOCl (30 mL)+17% EDTA (10 mL) Non chelators: Distilled water (40 mL) , 2.5% NaOCl (40 mL), Agitation system: NI, PUI Coronal, middle and apical	2.5% NaOCl+17% EDTA>Distilled water and 2.5% NaOCl
Naaman et al. 2007 (31)	Single rooted teeth	Sectioning and SEM (1500x) n=36	Chelators: 17% EDTA (3 mL), 50% CA (<1 mL) Non chelators: 5.25% NaOCl (3 mL), Agitation system: PUI Coronal, middle and apical	5.25% NaOCl+17% EDTA>5.25% NaOCl+50% CA
Neelakantan et al. 2017 (41)	Premolar (mand)	CBCT n=128	Chelators: 18 % EA (5 mL), 17% EDTA (5 mL) Non chelators: 6% NaOCl (5 mL), 3 % NaOCl (5 mL), Agitation system: NI, PUI, Finishing file, Navitip FX irrigation Whole canal	3% NaOCl+17% EDTA>16% NaOCl+18% EA>3% NaOCl
Rödig et al. 2010 (29)	Central and laterals (max)	Sectioning and microscope (30x) n=110	Chelators: 20% EDTA (20 mL), 10% CA (20 mL) Non chelators: 1% NaOCl (20 mL), water (20 mL), Agitation system: NI+files Coronal and apical	10% CA=20% EDTA=1% NaOCl+10% CA>1% NaOCl+20% EDTA>1% NaOCl and water (Apical groove) 20% EDTA=10% CA=1% NaOCl+10% CA=1% NaOCl+20% EDTA>1% NaOCl and Water (Coronal groove)
Salgado et al. 2009 (33)	Premolar (mand)	Sectioning and SEM (1000x) n=54	Chelators: EDTA (15 mL), 15% CA (15 mL), 17% EDTA-T (15 mL) Non chelators: 0.5% NaOCl (15 mL), Agitation system: NI, NI+MAF Coronal, middle and apical	17% EDTA-T+NaOCl (MAF)>15% CA=EDTA=17% EDTA-T>0.5% NaOCl (Coronal and middle third) 15% CA=17% EDTA-T+NaOCl (MAF)=EDTA=17% EDTA-T>0.5% NaOCl (apical)
Vineeta et al. 2014 (37)	Premolar (mand)	CBCT n=28	Chelators: 17% EDTA (2 mL)+Distilled water (1 mL), 0.2 % Chitosan (2 mL)+Distilled water (1 mL) Non chelators: Distilled water (1 mL), Agitation system: PUI Whole canal	Aqueous CH: 0.2% chitosan=17%EDTA>Distilled water Oil based CH: 0.2% chitosan>17% EDTA>Distilled water

CA: Citric acid, CBCT: Cone beam computed tomography, CH: Calcium hydroxide, EDTA: Ethylenediaminetetraaceticacid, EDTA-T: Ethylenediaminetetraaceticacid+ 0.2% lauryl sodium sulphate biologic detergent, MA: Maleic acid, MAF: Master apical file, mand: Mandibular, max: Maxillary, NaOCl: Sodium hypochlorite, NI: Needle irrigation, PA: Phosphoric acid, PAA: Peracetic acid, PUI: Passive ultrasonic irrigation, SAF- Self adjusting file, SEM: Scanning electron microscopy

**TABLE 2.** Characteristics of the included studies, which shows no difference between chelators and non-chelators

Author, year	Tooth	Method of assessment	Groups area assessed	Conclusion
Arslan et al. 2014 (36)	Premolar (mand)	Sectioning and Stereomicroscope and images (25x) n=48	Chelators: 17% EDTA (5 mL),7% MA (5 mL),10% CA (5 mL) Non chelators: 1% NaOCl (5 mL), Agitation system: NI Artificial groove (2-4.5 mm from apex only on one root canal wall of the split tooth)	7% MA=10% CA>17% EDTA=1% NaOCl
Bhuyan et al. 2015 (39)	Premolar (mand)	Sectioning and imaging (1000x) n=24	Chelators: 2.5% NaOCl (10 mL)+17% EDTA (5 mL) Non chelators: 2.5 % NaOCl (10 mL), Agitation system: F3 instrumentation Whole canal	2.5% NaOCl+17% EDTA=2.5% NaOCl
Çapar et al. 2014 (11)	Premolar (mand)	Sectioning and Stereomicroscope (30x) n=88	Chelators: 2.5% NaOCl (10 mL) + 17% EDTA (10 mL) Non chelators: 2.5% NaOCl (10 mL), Agitation system: NI, SAF, Endovac, PUI Artificial groove in apical region	2.5% NaOCl+17% EDTA=2.5% NaOCl (Only with NI,Endovac,PUI)
da Silva et al 2011 (34)	Molars (mand)	Sectioning and SEM (1000x) n=48	Chelators: 17% EDTA (5 mL), 10% CA (5 mL), 37% PA (5 mL) Non chelators: 2.5% NaOCl (5 mL), Agitation system: MAF Coronal, middle and apical	17% EDTA-T=37% PA>10% CA=2.5% NaOCl (Apical third)
Dias-Junior et al. 2021 (42)	Single rooted teeth	CLSM N=80	Chelators: 17% EDTA-T (6 mL),37% PA (6 mL) Non chelators: 2.5% NaOCl (6 mL), 70% ethanol (6 mL), Agitation system: NI, PUI Whole canal	70% ethanol>17% EDTA-T 70% ethanol>2.5% NaOCl (both)
Kuga et al. 2010 (20)	Central Incisors (mand)	Sectioning and SEM (1000x) n=40	Chelators: 17%EDTA (5 mL) Non chelators: 2.5% NaOCl (5 mL), Agitation system: Protaper instrument, K 3 endo instrument Cervical and apical	2.5% NaOCl=17% EDTA (Coronal and apical levels)
Lambrianidis et al. 1999 (30)	Single rooted teeth	Sectioning and imaging n=51	Chelators: 17% EDTA (10 mL) Non chelators: Sterile saline (10 mL), 3% NaOCl (10 mL), Agitation system: NI+Hand file Whole canal	17% EDTA=3% NaOCl (used as a final flush)
Tasdemir et al. 2011 (40)	Premolar (mand)	Sectioning and imaging (1000x) n=24	Chelators: 2.5% NaOCl (10 mL)+17% EDTA (5 mL) Non chelators: 2.5% NaOCl (10 mL), Agitation system: F 3 instrumentation Whole canal	2.5% NaOCl+17% EDTA=2.5% NaOCl

CA: Citric acid, CBCT: Cone beam computed tomography, EDTA: Ethylenediaminetetraaceticacid, LAI: Laser agitation irrigation, MAF: Master apical file, MA-Maleic acid, mand: Mandibular, max: Maxillary, NaOCl: Sodium hypochlorite, NI: Needle irrigation, PA: Phosphoric acid, SEM: Scanning electron microscopy

### Quality assessment

The Inter examiner reliability score between the two examiners was found to be 0.9, showing that the agreement was "almost perfect" (95% Confidence Interval: 0.83 to 0.96). The quality assessment of the included studies is shown in Table 3. Among the 17 included studies, 16 studies had "moderate" quality, and one study was categorised as "low" quality (30). The study by Lambrianidis et al. (30) was categorised as low quality of evidence since it had a score of less than 33% due to lack of true randomisation, sample size calculation, no inclusion of proper control group, no standardisation of samples, blinding of outcome assessors or equal baseline comparisons.

**TABLE 3.** Quality of included studies

	Abi-Rached et al. (10)	Anitha et al. (35)	Arslan et al. (36)	Bhuyan et al. (39)	Çapar et al. (11)	da Silva et al. (34)	Dias-Junior et al. 2021 (42)	Kuga et al. (20)	Kuştarcı et al. (38)	Lambrianidis et al. (30)	Li et al. (32)	Naaman et al. (31)	Neelakantan et al. (41)	Rödig et al. (29)	Salgado et al. (33)	Tasdemir et al. (40)	Vineeta et al. (37)
1. Was true randomisation used for assignment of teeth to treatment groups?	0	0	0	0	0	0	0	0	1	0	0	0	0	0	0	0	0
2. Was sample size calculated?	0	0	0	0	0	0	0	0	0	0	0	0	1	0	0	0	0
3. Were the samples standardised?	1	1	1	1	1	1	1	1	1	0	0	0	1	1	1	1	1
4. Was appropriate control group included in the study?	1	1	1	1	1	1	0	0	0	0	1	1	1	1	1	1	1
5. Were those delivering treatment blinded to treatment assignment?	0	0	0	0	0	0	0	0	0	0	0	0	0	0	0	0	0
6. Was baseline comparisons equal in all groups?	1	1	1	1	1	0	1	1	1	0	1	1	1	1	1	1	1
7. Was the outcomes assessed by multiple assessors?	1	0	1	0	1	1	0	1	0	0	0	1	0	1	1	0	0
8. Were the assessors blinded to the treatment assignment?	1	0	1	0	1	1	0	0	1	0	0	0	0	0	1	0	0
9. Were treatment groups treated identically other than the intervention of interest?	1	1	1	1	1	1	1	1	1	1	1	1	1	1	1	1	1
10. Were outcomes measured in the same way for treatment groups?	1	1	1	1	1	1	1	1	1	1	1	1	1	1	1	1	1
11. Were outcomes measured in a reliable way?	1	1	1	1	1	1	1	1	1	1	1	1	1	1	1	1	1
12. Was appropriate statistical analysis used	1	1	0	1	0	1	1	1	1	1	1	1	1	1	1	1	0
Total Score	9	7	8	7	8	8	6	7	8	4	6	7	8	8	9	7	6

### Influence of curvature on the removal of CH

Among the included studies, 5 studies were performed on teeth with curvature varying from 0° to 10o (34, 36, 38, 39, 40). Among these studies, 2 studies (39, 40) reported no beneficial effect of a chelator in removing calcium hydroxide. In contrast, one study (38) concluded chelator to be more efficacious in the removal of calcium hydroxide in comparison to non-chelator. Arslan et al. (36) reported maleic acid and citric acid alone to be more superior in removing CH from root canals with curvature <10°. da Silva et al. (34) used teeth with root canal curvature <5° and concluded chelators to be more superior in removing CH in the coronal and middle third of the root canals alone (Appendix 2).

### Role of chelators in the removal of calcium hydroxide with needle irrigation

A total of 7 studies (11, 32, 33, 36, 38, 41, 42) assessed the efficacy of chelator versus non-chelator in removing calcium hydroxide. Among which 4 studies (32, 33, 38, 41) concluded that the use of chelators significantly improved the removal of CH compared to non-chelators when delivered using needle irrigation technique. However, the studies comparing EDTA irrigation to ethanol (42) and NaOCl (11, 36) irrigation did not improve CH removal significantly.

### Role of chelators in the removal of calcium hydroxide with agitation systems

A total of 16 studies (10, 11, 20, 29-35, 37-42) had compared chelator versus non-chelators along with agitation techniques such as passive ultrasonic instrumentation and rotary instrument. Among the 16 studies, 9 studies (10, 29, 31-33, 35, 37, 38, 41) have found that chelators were more effective in removing calcium hydroxide when compared to non-chelators. However, 7 studies (11, 20, 30, 34, 39, 40, 42) showed that EDTA or citric acid irrigation is not superior to NaOCl irrigation in CH removal when used as an adjunct with various agitation techniques (Tables 1, 2).

## DISCUSSION

In the included 17 studies (10, 11, 20, 29-42), differences were observed in the following aspects: (a) chelators: volume, concentration, duration and delivery method, (b) Outcome assessed: methodology (sectioning-SEM, image analysis, CT, CBCT). Additionally, studies that used SEM for assessment had differences in magnification and scoring criteria, (c) Placement of CH: in longitudinal artificial grooves and (or) in intact root canals.

### Calcium chelators versus non-chelators in the removal of CH using needle irrigation

Based on the inclusion criteria, the literature search identified 7 studies (11, 32, 33, 36, 38, 41, 42) that compared the use of calcium chelators with non-chelators using needle irrigation to remove CH. All these studies (11, 32, 33, 36, 38, 41, 42) assessed the efficiency of EDTA in removing CH in comparison to NaOCl, of which 4 studies (32, 33, 38, 41) with moderate quality of evidence have proven that the use of EDTA produces significantly superior removal in comparison to that of a non-chelator even with needle irrigation. Similarly, 2 studies (33, 36) with moderate quality have assessed the efficiency of citric acid in removing CH compared to that of NaOCl when used with needle irrigation and have proven that citric acid is significantly superior in removal efficiency. In addition, studies done by Arslan et al. (36) and Neelakantan et al. (41) revealed that maleic acid and etidronic acid have significant superior removal efficiency compared to non-chelators. The significant increase in the removal of CH by chelators could be due to their ability to chelate calcium in the presence of water or any other vehicle facilitating the ease of removing the medicament (43). The cumulative evidence of the studies mentioned above shows that chelators significantly improved the removal of CH compared to non-chelators when delivered using the needle irrigation technique.

### Calcium chelators versus non-chelators in CH removal using irrigation agitation or instrumentation methods

The literature search identified 5 studies with moderate quality of evidence (31, 32, 35, 37, 41) which compared the use of calcium chelators with non-chelators along with PUI for removal of CH and concluded that chelators with PUI as more superior. The probable reason could be the synergistic effect of calcium chelators with the acoustic streaming and cavitation produced by the ultrasonic agitation inside the root canal (44). Similarly, 5 studies (10, 11, 33, 34, 41) with moderate quality showed significant superiority of calcium chelators over non-chelator in the removal of CH when used along with agitation of rotary or hand files. However, 3 studies with moderate quality (29, 38, 40) showed no difference between the use of NaOCl alone or along with EDTA when used with hand files as agitation. The reason might be attributed to the formulation of CH used and the assessment method (sectioning and scoring criteria under magnification or sectioning and imaging under magnification) used for CH removal. A study done by Kuga et al. (20) showed that EDTA did not enhance the removal of CH compared to that of NaOCl when used along with rotary files. This is probably due to the use of propylene glycol as the vehicle for CH medicament. It has been shown that vehicles like silicone oil and methylcellulose used in CH affects the retrieval (30, 43). This review favoured the use of calcium chelators to enhance the removal of CH when used along with endodontic file agitation. Irrigation agitation techniques such as SAF (11), Navi tip (41) and laser agitation system (38) were found to be superior in CH removal when compared to a non-chelator. However, in the current review, only one study has been included for SAF (11), Navi tip (41) and laser agitation (38) technique based on selection criteria. Future research can be planned to assess the role of these dynamic agitation techniques in CH removal. Hence, the current evidence shows that calcium chelators effectively remove CH using irrigation agitation or instrumentation techniques compared to a non-chelator. 

### Role of a vehicle in the removal of CH

The vehicle used for the CH intracanal medicament can also affect the ease of removal. The vehicles mixed with CH powder play an important role in the overall dissociation process because they determine the velocity of ionic dissociation, causing the paste to be solubilised and resorbed at various rates by the periapical tissues and from within the root canal (45). From the included studies, it can be concluded that the powder form of CH, mixed with sterile saline or distilled water, is more effectively removed with chelators when compared to a non-chelating agent.

### Limitations

Although all the included studies mentioned randomised allocation, only one study described the randomisation methods and allocation concealment used (38). Also, only some studies performed blinding (10, 11, 33, 34, 36, 38). This might increase the risk of bias; therefore, the interpretation of results must be made cautiously. Differences were observed in the methodology used to assess the outcome (CH removal). For example, different teeth and different areas of the teeth (coronal vs middle vs apical). Due to the inherent heterogeneity of the included articles, a meta-analysis could not be performed. Publications in the English language alone were included in this review. 

## CONCLUSION

Within the limitations of the current review, calcium chelators enhance the removal of the CH from the root canal system over non-chelators when used with needle irrigation technique, hand files, passive ultrasonic instrumentation and rotary instrument agitation. In addition, the powder form of CH, which is mixed with sterile saline or distilled water, is more effectively removed with chelators when compared to a non-chelating agent.

## Appendix

**APPENDIX 1.** Search criteria

Database	Search strategy
PubMed	(“calcium hydroxide”[All Fields] OR “intracanal medicament”[All Fields] OR medicament[All Fields]) AND (“irrigation solution”[All Fields] OR irrigating[All Fields] OR irrigated[All Fields] OR irrigant[All Fields] OR irrigate[All Fields] OR “chelating agents”[All Fields] OR “chelator”[All Fields] OR “citric acid”[All Fields] OR “maleic acid”[All Fields] OR “edetic acid”[All Fields] OR “edta” [All Fields] OR “phytic acid”[All Fields] OR “peracetic acid”[All Fields] OR “sodium hypochlorite”[All Fields])) AND ((removal[All Fields] OR retrieval[All Fields]) OR elimination[All Fields])
Scopus	(TITLE-ABS KEY (calcium hydroxide OR medicament OR ''intracanal medicament'') AND (TITLE-ABS-KEY (remove* OR elimination* OR retrieval*) AND (TITLE-ABS KEY (irrigation AND solution OR irrigating OR irrigated OR irrigant OR irrigate OR CHelator OR citric AND acid OR maleic AND acid OR EDTA OR phytic AND acid OR peracetic AND acid OR sodium AND hypoCHlorite)
Proquest	ti(calcium hydroxide’’ OR medicament OR ‘‘intracanal medicament’’) AND ti(removal OR elimination OR retrieval) AND ti (irrigation OR irrigating OR irrigated OR irrigant OR irrigate OR Chelators OR citric NEAR acid OR maleic NEAR acid OR EDTA OR phytic NEAR acid OR peracetic NEAR acid OR sodium hypochlorite)
Cochrane	'Irrigation OR irrigating OR irrigated OR irrigant OR irrigate OR Chelators OR citric NEAR acid OR maleic NEAR acid OR EDTA OR phytic NEAR acid OR peracetic NEAR acid OR sodium hypochlorite in Title Abstract Keyword AND calcium hydroxide OR medicament OR intracanal me dicament in Title Abstract Keyword AND removal OR retrieval OR elimination in Title Abstract Keyword
EMBASE	(calcium hydroxide or medicament or intracanal medicament).mp. [mp=title, abstract, heading word, drug trade name, original title, device manufacturer, drug manufacturer, device trade name, keyword, floating subheading word, candidate term word] AND (removal or retrieval or elimination).mp. [mp=title, abstract, heading word, drug trade name, original title, device manufacturer, drug manufacturer, device trade name, keyword, floating subheading word, candidate term word] AND (irrigation or irrigating or irrigated or irrigant or irrigate or Chelators or citric acid or maleic acid or EDTA or phytic acid or peracetic acid or sodium hypochlorite).mp. [mp=title, abstract, heading word, drug trade name, original title, device manufacturer, drug manufacturer, device trade name, keyword, floating subheading word, candidate term word]
Google	(calcium hydroxide OR medicament OR intracanal medicament) AND (removal OR elimination OR retrieval) AND (Chelators OR irrigants OR irrigant OR irrigate OR citric acid OR maleic acid OR EDTA OR phytic acid AND sodium hypochlorite

**APPENDIX 2.** Characteristics of included articles that assessed the influence of curvature on the removal of CH

Author, year	Tooth	Assessment method	Curvature	Groups area assessed	Conclusion
Arslan et al. 2014 (36)	Premolar (mand)	Sectioning and Stereomicroscope (25x) n=48	<10°	Chelators: 17% EDTA (5 mL), 7% MA (5 mL), 10% CA (5 mL) Non chelators: 1% NaOCl (5 mL), Agitation system: NI Artificial groove in apical region	7% MA=10% CA>17% EDTA=1% NaOCl
da Silva et al. 2011 (34)	Molars (mand)	Sectioning and SEM (1000x) n=48	≤5°	Chelators: 17% EDTA (5 mL), 10% CA (5 mL), 37% phosphoric acid (5 mL) Non chelators: 2.5% NaOCl (5 mL), Agitation system: MAF Coronal, middle and apical	37% PA=10% CA=17% EDTA-T>2.5% NaOCl (Coronal and middle third) 17% EDTA-T=37% PA>10% CA=2.5% NaOCl (Apical third)
Kuştarcı et al. 2016 (38)	Premolar (mand)	Sectioning and Stereomicroscope (6x) n=160	<10°	Chelators: 17% EDTA (6.5 mL), Qmix (6.5 mL), 1% PAA (6.5 mL) Non chelators: 2.5% NaOCl (6.5 mL), Agitation system: NI, LAI Artificial groove (2-6 mm from apex only)	LAI>NI 17% EDTA=Qmix=1% PAA>2.5% NaOCl (for both LAI and NI)
Bhuyan et al. 2015 (39)	Premolar (mand)	Sectioning and imaging (1000x) n=24	<10°	Chelators: 2.5% NaOCl (10 mL)+17% EDTA (5 mL) Non chelators: 2.5 % NaOCl (10 mL), Agitation system: F3 instrumentation Whole canal	2.5% NaOCl+17% EDTA=2.5% NaOCl
Tasdemir et al. 2011 (40)	Premolar (mand)	Sectioning and imaging (1000x) n=24	<10°	Chelators: 2.5% NaOCl (10 mL)+17% EDTA (5 mL) Non chelators: 2.5% NaOCl (10 mL), Agitation system: F 3 instrumentation Whole canal	2.5% NaOCl+17% EDTA=2.5% NaOCl

CA: Citric acid, CBCT: Cone beam computed tomography, CH: Calcium hydroxide, EDTA: Ethylenediaminetetraaceticacid, EDTA-T: Ethylenediaminetetraaceticacid+ 0.2% lauryl sodium sulphate biologic detergent, MA: Maleic acid, MAF: Master apical file, mand: Mandibular, max: Maxillary, NaOCl: Sodium hypochlorite, NI: Needle irrigation, PA: Phosphoric acid, PAA: Peracetic acid, PUI: Passive ultrasonic irrigation, SAF: Self adjusting file, SEM: Scanning electron microscopy
